# Crooked nose: outcome evaluations in rhinoplasty

**DOI:** 10.1590/S1808-86942011000400016

**Published:** 2015-10-19

**Authors:** Lisandra Megumi Arima, Leandro Castro Velasco, Romualdo Suzano Louzeiro Tiago

**Affiliations:** 1 3^rd^-year resident physician (R3) - ENT Department - HSPM; 2 3^rd^-year resident physician (R3) - ENT Department - HSPM; 3 PhD in Sciences - Graduate Program in Otorhinolaryngology and Head and Neck Surgery - Federal University of São Paulo. Post doctorate from the Federal University of São Paulo. Attending ENT at the HSPM

**Keywords:** outcome assessment (health care), patient satisfaction, quality of life, rhinoplasty

## Abstract

**Abstract:**

A crooked nose is the result of deformities that might involve the bony nasal pyramid, the upper and lower lateral cartilages, and nasal septum, causing complaints of aesthetic and/or functional nature.

**Purpose:**

To evaluate how satisfied are those patients who underwent rhinoplasty to correct crooked nose, through the questionnaire Rhinoplasty Outcomes Evaluation (ROE).

**Material and method:**

A longitudinal study with retrospective analysis of preoperative satisfaction and prospective analysis of postoperative satisfaction of patients who underwent rhinoplasty. ROE questionnaire was applied twice in the same visit aiming at measuring patient satisfaction in both pre and postoperative periods. Nineteen patients who underwent rhinoplasty answered the ROE.

**Results:**

For all patients who underwent rhinoplasty, the average preoperative satisfaction score was of 24.6±11.3, while the average postoperative score was of 76.1±19.5 (*p*<0.0001). Average differences between pre and postoperative satisfaction scores in patients younger than 30 years of age were lower than those reported by ≥30-year-old patients (*p*=0.05).

**Conclusion:**

From the Rhinoplasty Outcomes Evaluation questionnaire, it is possible to demonstrate the impact that rhinoplasty to correct a crooked nose determines the quality of life of patients. Approximately 90% of patients undergoing rhinoplasty believed they achieved a good or excellent postoperative result.

## INTRODUCTION

Rhinoplasty has become one of the main cosmetic surgeries performed by otorhinolaryngologists and plastic surgeons. The major indications for rhinoplasty are: cosmetic and cosmetic-functional. Cosmetic-functional rhinoplasty, or rhino-septoplasty, means the cosmetic repair of the nasal pyramid, together with surgery of the nasal septum in order to improve patient complaints associated with nasal obstruction and hyposmia. In those cosmeticonly procedures, the physician must assess the reason for which the patient wishes to be submitted to the procedure. Often times, the reason involves the need to please other people, social or professional ambition; the surgeon then has a great responsibility, which is to accept or refuse the patient's request^1^. Pre and intraoperative planning are essential in order to achieve good results; the surgeon must carefully examine the nose in order to determine which pathological condition there is and which surgical procedure is needed^2^.

The crooked nose is a generic term used to define all deformities which involve the nasal pyramid deviation in relation to the facial medio-sagittal plane^3^. The crooked nose is the result of complex deformities which may involve the bony nasal pyramid, the upper and lower lateral cartilages and, especially, the nasal septum, leading to cosmetic and functional complaints^4^. The crooked nose's major component is the extremely deviated nasal septum^5^. Therefore, in order to correct the crooked nose, the nasal septum must be the treatment's target. Even in the absence of obstructive complaints, small septum deviations may impact proper nasal alignment^6^. Therefore, it is important to have a broad knowledge of the nasal anatomy and the external and internal forces which act on these structures so as to employ the many existing surgical techniques^5^. Congenital and trauma causes, and those associated with previous nasal surgeries may be present in the patient's history^2^,^5^, ^6^, ^7^, ^8^.

Most of the papers discussing cosmetic surgery bear discussions regarding surgical techniques, access pathways, complications, sequelae and reoperation rates^2^,^4^, ^5^, ^6^, ^7^, ^8^, ^9^, ^10^, ^11^, ^12^. The assessment of the intervention's final result was not very much studied under the patient's viewpoint, and such analysis is very important because patient satisfaction is the prevailing factor for surgical success^13^, ^14^, ^15^, ^16^, ^13^.

In the recent decade, numerous have been the papers published in order to validate a reliable questionnaire to be employed in patients submitted to cosmetic surgery, with the goal of measuring patient satisfaction after the procedure^17^, ^18^, ^19^, ^20^, ^21^, ^22^. Some instruments, such as questionnaires, which assess quality of life and self-image have become a gold standard and came to replace the simplistic way used to ask the patient whether or not he/she had noticed any improvement after surgery^23,^^24^.

The use of broadly accepted questionnaires brings about great advantage, because it standardizes assessment and enables a comparison of different techniques; besides, it helps measuring the positive and negative effects, and to identify possible patients who may not benefit from surgery^13^.

Alssarraf et al. tested and supplied an assessment tool for numerous facial cosmetic procedures, including rhinoplasty, with reliability, internal consistency and method validity^18,^^19^. This questionnaire is a tool the surgeon may have in order to objectively assess some qualitative variables associated with the cosmetic surgery, such as psychological, social and emotional aspects^18,^^19,^^25^.

## OBJECTIVE

To assess the satisfaction of patients submitted to rhinoplasty to correct a crooked nose based on the *Rhinoplasty Outcomes Evaluation* (ROE) Questionnaire.

## MATERIALS AND METHODS

We studied 35 consecutive patients submitted to rhinoplasty to correct a crooked nose, with an endonasal approach. The surgeries were carried out in the Otorhinolaryngology Department of a tertiary hospital in the city of São Paulo (SP) between January of 2002 and July of 2009. The procedures were either done or supervised by the third author.

We included all the patients submitted to rhinoplasty in order to correct a crooked nose, with 12 months to 8.4 years of postoperative follow up. Patients from 17 years of age and up, had agreed to the procedure and signed a free and informed consent form when they came to our institution after telephone contact.

We excluded those patients with whom it was not possible to make telephone contact, those who did not agree with the consent form, and those who did not come to the interview (Table 1).

**Table 1:** Reasons why and number of patients taken off the sample.

Reason	N
It was not possible to get in touch with the patient	11
Wrong telephone number	10
Did not answer the phone	1
Did not come	5
Despite the contact and the appointment setup	4
Could not come to the hospital at the time of the data collection	1
TOTAL	16

We carried out a longitudinal, retrospective, cohort study, with a retrospective analysis of preoperative satisfaction and a prospective one concerning the postoperative satisfaction. The patients were invited by telephone and came to the institution where the surgical procedure was carried out in order to answer the ROE questionnaire, translated into Portuguese^18,^^19^. Those patients who came to the hospital, received information in regards of the study and agreed to participate in the study through the Informed Consent Form. The project was evaluated and approved by the Ethics in Research Committee of the institution (Approval #20/2010).

The ROE questionnaire was employed twice during the same visit, with the goal of measuring patient satisfaction before and after surgery. The preoperative answers were based on the pictures taken in a standardized way before the surgical procedure. The post-operative responses were based on the current patient status^22,^^26^.

Alssarraf et al. tested and validated this tool (ROE), which aims at, by using six questions, assessing the quality of life in three subjective realms: physical, mental/emotional and social (Chart 1)^19^.

Chart 1 **Rhinoplasty Outcomes Evaluation (ROE) questionnaire.**

1) How much do you like the appearance of your nose?
2) How much can you breathe through your nose?
3) How much do you think your friends and those close to you like your nose?
4) Do you think the appearance of your nose limits your social or professional activities?
5) How safe are you that your nose has the best possible appearance?
6) Would you like to surgically change the appearance or function of your nose?

Each question in the questionnaire was answered with scores within a scale between zero and four (zero being the most negative answer, and four being the most positive one). In order to reach the final result in the scale, we added up the responses from each question, and such result was divided by 24 and multiplied by 100 - from that we obtained a value which varied between zero and 100 (zero represents minimum satisfaction and 100 the maximum one)^19^. The final result was then divided in classes, according to quartiles: zero to <25 and 25 to <50 (failure); 50 to <75 (good); and ≥75 (excellent). The class division in a scale between zero and 100 can be done in groups of 25, which are given the name of quartiles. Those patients within the second and third quartiles are in the center of the distribution, and the remaining 50% are further divided into two 25% parts, one to the left, called subnormal or first quartile; and another to the right, called supernormal or fourth quartile^27^.

After data collection, we obtained three variables: satisfaction score that the patient had with his/her own image before surgery; satisfaction score with the current result; and the difference between the pre and postoperative satisfaction scores. We surveyed the data concerning: age, gender, and postoperative follow up. The data was plotted in an electronic spreadsheet using the Microsoft Excel (Microsoft Corporation) software.

For data statistical analysis, we used: the t-paired test (to compare the mean scores between pre and postoperative) and the Mann-Whitney non-parametric test (to compare the mean values of the satisfaction scores between the pre and postoperative scores of those patients submitted to rhinoplasty to correct a crooked nose according to age and follow up time. A *p* value ≤0.05 was considered statistically significant.

## RESULTS

The initial sample had 35 patients, and 19 of these answered the questionnaire. The reasons for not answering it, given by the remaining patients are depicted on Table 1.

The mean age of the 19 patients who participated in the study was 37.9 years; 17 (89.5%) were males and two (10.5%) were females.

**Figure 1:**
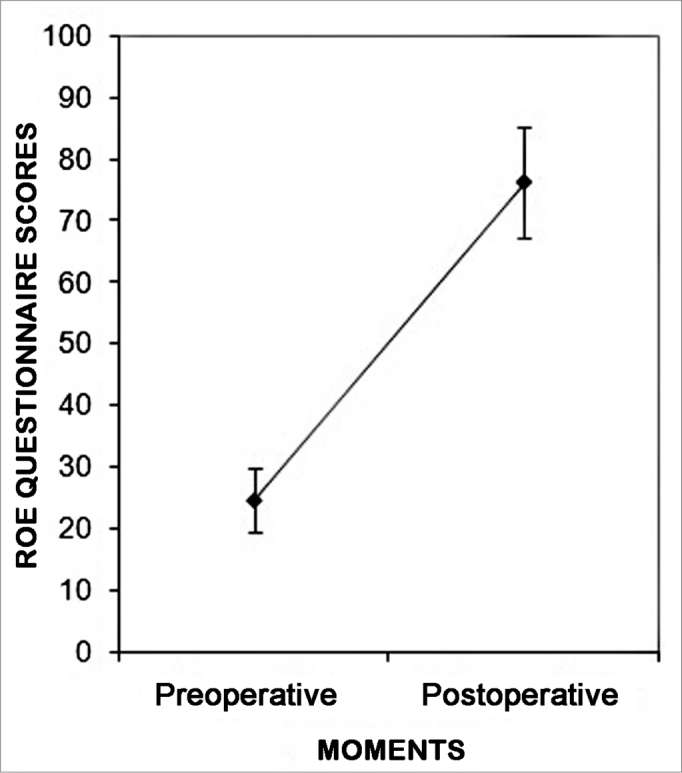
Pre and postoperative satisfaction score mean values from the patients submitted to rhinoplasty to correct a crooked nose (Mean ± 1SD). t-paired test *p* 0.0001.

The mean satisfaction score from all the patients submitted to rhinoplasty to correct a crooked nose in the pre-op was 24.6 ± 11.3 and in the post-op it went up to 76.1 ± 19.5 (Figure 1). There was a difference between the mean scores in the post and preoperative of 51.5 (*p*<0.0001).

In the preoperative we noticed that 18 (94.7%) patients had satisfaction rates <50, with only one patient (5.3%) who had it between 50 and <75. In the postoperative we noticed that 84.2% moved from the <50 class to the 50 to <75 considering it a good result (21.1%); and ≥75 considered it an excellent result (68.4%). Although 10.6% (5.3% + 5.3%) of the patients remained in the same class, there was no worsening in the initial situation (Table 2). In the post-op we noticed that 100% of the patients had an increase in the pre and postoperative scores, in other words, in none of the patients the satisfaction score in the postoperative time was lower than that from the preoperative time.

**Table 2:** Absolute and relative frequency of the patients submitted to rhinoplasty to correct a crooked nose according to their satisfaction rate in the pre (PRE) and postoperative (POST) times.

	Moment			PRE		
			<25	25 to <50	50 to <75	Total
	25 to <50	N	1	1	0	2
	(failure)	%	5,3%	5,3%	0,0%	10,5%
POST	50 to <75	N	3	0	1	4
	(good)	%	15,8%	0,0%	5,3%	21,1%
	≥75	N	6	7	0	13
	(excellent)	%	31,6%	36,8%	0,0%	68,4%
		N	10	8	1	19
	Total	%	52,6%	42,1%	5,3%	100,0%

As far as their ages are concerned, the sample was divided in two classes: <30 years; and ≥30 years. We noticed that age was a factor which influenced the mean difference among satisfaction scores between post and preoperative, in other words, those patients <30 years had less satisfaction increment when compared to those ≥30 years (*p*=0.05), as depicted on Table 3.

**Table 3:** Mean difference of the pre and postoperative scores of those patients submitted to rhinoplasty in order to correct a crooked nose, according to age.

Crooked Nose	<30 years	≥30 years	Mann-Whitney test (*p*)
Mean	38,1	59,4	
Standard deviation	24,3	20,1	0,05
N	7	12	

The mean follow up time after rhinoplasty was 40.9 months, which varied from 12 months to 8.4 years. The sample was broken down into two classes according to the follow up period: 12 to <60 months; and ≥60 months.

The mean values of the differences found in satisfaction rates between pre and postoperative according to follow up time were similar in the two classes, without statistically significant differences (Table 4).

**Table 4:** Mean value of the pre and postoperative satisfaction scores of patients submitted to rhinoplasty to correct a crooked nose according to follow up time.

Crooked Nose	12 to <60 months	≥60 months	Mann-Whitney test (*p*)
Mean	56,1	34,4	
Standard deviation	19,0	33,9	0,22
N	15	4	

## DISCUSSION

Some factors may influence patient satisfaction, such as culture, life experience and, mainly, the patient's expectations in relation to the final result, which may or may not be realistic.^22,^^24^ Although, often times the procedure may be considered a success by the surgeon, the patient may feel not pleased with it, and the opposite is also true. Therefore, it is important for the surgeon to understand the patient's complaints, and to analyze the proportions and relationships between the nose and the face through physical exam and photographic documentation.^26^

The method hereby utilized, a retrospective assessment of patient preoperative satisfaction, and prospective evaluation of the patient's postoperative satisfaction, was similar to the one published by other authors.^22^ In the study published by Hellings et al. the preoperative satisfaction scores were based on the patient's memory.^22^ In the present investigation, the preoperative satisfaction scores were based on the patient's memory and in the photographies recorded in a standardized fashion during the preoperative time. In other words, when answering the questionnaire, the patient had the support from the preoperative pictures. There are very few published papers which used the ROE questionnaire, and the other two studies which used it, did so in a prospective way^17,^^19^.

One of the main success factors for surgery is the patient's satisfaction after the procedure. Recently, many review papers were carried out in order to select a tool capable of measuring and analyzing patient satisfaction in the post-op^13^, ^14^, ^15^, ^16^, ^17^, ^18^, ^19^, ^20^, ^21^, ^22^.

Alssarraf et al. created and validated the ROE Questionnaire, a tool of easy and fast application, useful to assess different types of patients, approaches and surgical techniques^17,^^19,^^22^. In the present study, this questionnaire was used in order to measure the satisfaction of the patients submitted to rhinoplasty in order to correct a crooked nose.

The correction of a crooked nose (bony and/or cartilaginous pyramid) still remains a challenge. The natural force existing in the cartilaginous structures and soft tissue (shortened muscles and connective tissue on the crooked side) continue to act on the nose submitted to rhinoplasty and makes it difficult to achieve an excellent result in the postoperative time. Another factor which may cause the nose to return to its crooked shape is the incomplete correction of the deviated nasal septum^3,^^9,^^10^. In our sample, septoplasty associated with rhinoplasty was done in 90% of the patients, a similar result to another study in which 89% of the patients were submitted to septoplasty^6^.

The 11 patients who did not participate in the study because of difficulties in contacting them (wrong number or did not answer the phone) represented a random loss. Nonetheless, the five patients called and who did not show up could change the mean score because of a greater or lower satisfaction rate. The no-show may be associated with a happy patient who is no longer interested in going back for reassessments.

All the 19 procedures to correct crooked noses were done from an endonasal approach. The use of the external or open approach for direct visualization of the anatomic structures involved in the crooked nose is preferred by many authors, especially when the defect is considered severe^2^, ^3^, ^4^, ^5^, ^6^, ^7^, ^8^, ^9^, ^10^, ^11^, ^12^,^12^. Jang et al. carried out a study in order to classify the types of crooked nose, and obtained a failure rate of 11% among operated patients, and the non-satisfactory result seen in 50% of these cases was attributed to the difficulty in exposure because of the conservative endonasal approach^12^. It was noticed that the endonasal approach was not a limiting factor for the results presented in the present study (Table 2), having seen no worsening in the initial situation of any of the patients; and 89.5% had good (21.1%) or excellent (68.4%) post-op results. The patients submitted to rhinoplasty may require a second-look surgery in order to correct post-operative deformities (revision rhinoplasty), in about 2 to 5% of the cases^28^. In cases of trauma or crooked noses, this rate may be higher^28^. Therefore, the type of surgical approach, endonasal or open, may not be a factor which impacts the non-satisfactory result and the rate of revision procedures.

The postoperative score, in all the patients submitted to rhinoplasty to correct a crooked nose was higher than their preoperative score. The mean difference between the post and preoperative scores was 51.5 (76.1 in the postoperative and 24.6 in the pre-op), higher than the results published by Alssarraf et al. of 44.5 (83.3 in the post-op and 38.8 in the pre-op)^19^, which assessed patients submitted to rhinoplasty, regardless of the surgical technique utilized. The greater difference between the post and preoperative values seen in the present study is due to a lower preoperative mean value arising from a greater number of patients with functional complaints. According to Hellings et al., patients who need two or more procedures have a mean score difference between post and pre-op of 16 (58.8 in the post-op; and 42.8 in the preoperative),^22^ which shows the difficulty in achieving an excellent result in patients submitted to many surgical procedures.

The classification in quartiles helps the surgeon define which are the patients who can most benefit from the rhinoplasty. Those patients in the first and second quartiles in the preoperative time are the individuals who are not happy with the appearance and function of their noses; therefore, these are the ones which may have a great benefit from the procedure. The patients who, in the pre-op, are in the third and fourth quartiles are the ones who do not have major cosmetic and/or functional complaints and may not obtain significant improvements in the postoperative, or even run the risk of getting worse than their original condition. In the present study we tried to define the first and second quartiles in the postoperative, as non-satisfactory result or failure, having seen that the patients who are within this range are not happy with their appearance. Most of the patients in the third quartile are pleased with the rhinoplasty, which was considered a good result. The patients in the fourth quartile are hugely pleased with the result.

Upon analyzing the reason why two patients kept post-operative satisfaction <50 (failure), it was noticed that both cosmetics and function remained as complaints after the surgical procedures. The term “failure” is used in order to express patient dissatisfaction with the result. The patient who reported a preoperative score within the 50 to <75 range, remained in the same class (good result) after surgery (Table 2). This patient had an asymmetry in the lateral branch of the wing cartilages, and such characteristic is an anatomical limitation which made it difficult to have cosmetic improvements.

Younger patients have higher expectations in relation to the final cosmetic result, probably because of greater peer pressure, with difficulties to accept changes to their self-images^20^. In our study, we noticed a statistically significant difference (*p*=0.05) between the pre and postoperative satisfaction score mean values for the age classes <30 years and ≥30 years (Table 3). Therefore, the younger patients submitted to rhinoplasty to correct a crooked nose require a more detailed preoperative education, with information concerning the limitations of the procedure in reaching satisfactory results.

The final result from the rhinoplasty can be seen after 12 months of follow up. In the present study we noticed that the patients who had a longer postoperative follow up (≥60 months) had pre and postoperative mean score differences similar to those patients who were operated in the last 60 months (Table 4). Therefore, a five-year postoperative follow up for rhinoplasty may be enough to assess long term results.

The ROE Questionnaire is a tool which enables the assessment of results from different surgical techniques used to correct nasal deformities. Although the crooked nose correction requires the use of more elaborated techniques, our study showed that 68.4% of patients submitted to rhinoplasty to correct a crooked nose obtained excelled postoperative results (satisfaction ≥75), as depicted on Table 2. Okur et al., analyzed deviated angle measures from an objective method (result quantification based on a computer program), and considered that the closer the postoperative angle was from the ideal angle, the greater was the surgery's success rate^3^. They reported that 66.7% of the patients reached good or excellent results (result ≥70)^3^. So far, the literature has no paper describing result assessment utilizing the ROE Questionnaire in patients submitted to rhinoplasty to correct a crooked nose.

## CONCLUSION

Based on the *Rhinoplasty Outcomes Evaluation* Questionnaire, it is possible to show the impact rhinoplasty, done to correct a crooked nose, has on the patient's quality of life. Approximately 90% of the patients submitted to rhinoplasty to correct a crooked nose believe they had a good or excellent postoperative result.
